# Sagittal knee kinematics in relation with the posterior tibia slope during jump landing after an anterior cruciate ligament reconstruction

**DOI:** 10.1186/s40634-020-00289-9

**Published:** 2020-09-21

**Authors:** Michèle N. J. Keizer, Juha M. Hijmans, Alli Gokeler, Egbert Otten, Reinoud W. Brouwer

**Affiliations:** 1 University of Groningen, University Medical Center Groningen, Center for Human Movement Sciences, FA 23 – PO Box 219, Groningen, 9713 AV The Netherlands; 2 Department of Rehabilitation Medicine, University of Groningen, University Medical Center Groningen, Groningen, The Netherlands; 3 Luxembourg Institute of Research in Orthopaedics, Sports Medicine and Science (LIROMS), Luxembourg, Luxembourg; 4 Department Exercise & Health, Exercise Science and Neuroscience, University of Paderborn, Paderborn, Germany; 5 Department of Orthopaedic Surgery, Martini Hospital Groningen, Groningen, The Netherlands

**Keywords:** Knee, In-vivo knee kinematics, Anatomy, Tibia plateau

## Abstract

**Purpose:**

An increased posterior tibia plateau angle is associated with increased risk for anterior cruciate ligament injury and re-rupture after reconstruction. The aims of this study were to determine whether the tibia plateau angle correlates with dynamic anterior tibia translation (ATT) after an anterior cruciate ligament reconstruction and whether the tibia plateau angle correlates with aspects of knee kinematics and kinetics during jump landing.

**Methods:**

Thirty-seven patients after anterior cruciate ligament reconstruction with autograft hamstring tendon were included. Knee flexion angle and knee extension moment during single leg hops for distance were determined using a motion capture system and the dynamic ATT with its embedded method. The medial and lateral posterior tibia plateau angle were measured using MRI. Moreover, passive ATT was measured using the KT-1000 arthrometer.

**Results:**

A weak negative correlation was found between the maximal dynamic ATT and the medial tibia plateau angle (*p* = 0.028, r = − 0.36) and between the maximal knee flexion angle and the lateral tibia plateau angle (*p* = 0.025, r = − 0.37) during landing. Patients with a smaller lateral tibia plateau angle show larger maximal knee flexion angle during landing than the patients with larger lateral tibia plateau angle. Also, the lateral tibia plateau angle is associated the amount of with muscle activity.

**Conclusion:**

The posterior medical tibia plateau angle is associated with dynamic ATT. The maximal knee flexion angle and muscle activity are associated with the posterior lateral tibia plateau angle.

**Level of evidence:**

III

## Background

The posterior tibia plateau angle (PTPA) is defined as the angle of the posterior tibia plateau relative to the plane orthogonal to the longitudinal axis of the tibia in the sagittal plane. The PTPA has a medial angle (MPTPA) and a lateral angle (LPTPA). On radiographs, the PTPA has shown to be increased in patients who have had an ACL injury compared to a group that had no injury [34]. Therefore, increased PTPA is a risk factor for anterior cruciate ligament (ACL) injury [18, 29, 33] and re-rupture after an ACL reconstruction [10, 15, 36].

A correlation between the PTPA and knee kinematics using cadaveric experiments (e.g. [13]) and model studies (e.g. [21, 30, 32]) have been found: it has been reported that an increased PTPA is associated with larger calculated anterior tibia translation (ATT) [30] and an increased passive ATT (ATTp) in ACL injured [28], ACL reconstructed (ACLR) [5] and cadaveric knees [13]. In cadaveric knees, 80% to 90% of the anteriorly applied tibial loads, using the drawer test, is supported by the ACL [4]. As a larger PTPA is associated with an increase in ATTp, some orthopaedic surgeons consider and recommend a combined ACLR and anterior closing wedge tibial osteotomy in patients after an ACL injury. The PTPA may play a significant role in the force load on the ACL. On biomechanical grounds patients who have a large PTPA may show different dynamics of the knee, due to a difference in direction of the condylar reaction force. Moreover, it is found that there is a correlation between the PTPA and the knee moment by using a model of a drop vertical jump [2]. As far as known to the authors it is not yet known if and how the PTPA correlates with dynamic in-vivo kinematics and kinetics during high demanding functional tasks, such as jump landing, after an ACLR.

This study sought to determine whether the PTPA (MPTPA and LPTPA) positively correlate with the dynamic ATT (ATTd) after an ACL reconstruction during a jump landing. The second aim was to determine how the MPTPA and LPTPA correlates with the knee flexion angle and knee internal extension moment during landing. We hypothesized that the PTPA is positively correlated with the maximal ATTd during jump landing and that the PTPA does have a positive correlation with the knee flexion angle and internal extension moment of the knee.

## Methods

The study was conducted at the … in the period from April 2018–November 2019. The study design, procedure, and protocol are approved by the Medical Ethical Committee of the …. (METC number: 2017.658). All participants were informed about the procedures and aim of the study by letter and they signed an informed consent before the start of the measurement.

### Participants

Sample size estimations were performed a priori. Means and standard deviations from available data from the literature [19] were entered for the MPTPA in correlation with ATTp (r = 0.41). As no information about the correlation between ATTd and PTPA is available in the literature, the correlation between ATTp and MPTPA was used for the power analysis. Based on a statistical power set at α ≤ 0.05 and a power of 80% to detect a statistically significant correlation, 33 subjects were needed.

To be on the safe side of the statistical power, 37 patients (13 woman and 24 men; age: 18–39) were included in the study. Inclusion criteria were 1 to 2 years post-surgery, age between 18 and 45 years and ACLR with autograft hamstring tendon. Exclusion criteria were patients with cartilage pathology that needed concomitant surgical treatment and changed the standard rehabilitation, who underwent a revision ACLR, osteotomy or contralateral ACLR. For baseline characteristics see Table 1.

**Table 1:** baseline characteristics

	Mean	Range		Man	Woman
Age (year)	25.9	18–39	Gender	24	13
Height (cm)	181.7	161–197.6			
Weight (kg)	78.2	51.7–111.6		Yes	No
Month post-surgery	16.4	12–24	RTS/not RTS	26	11
Hop distance (cm)	132.6	37.4–183			
MPTPA (deg)	3.7	−2.1-8.8			
LPTPA (deg)	5.3	0.6–12.2			
Maximal dynamic ATT (mm)	12.1	−0.1-22.3			
KT-1000 arthrometer (mm)	5.1	1.1–9.5			

*MPTPA* medial posterior tibia plateau angle, *LPTPA* lateral posterior tibia plateau angle, *ATT* anterior tibia translation, *RTS* return to sports

### Surgical technique

Subjects were included in this study after surgery, though in all patients the same surgical procedures were followed. For all surgical procedures, ipsilateral gracilis and semitendinosus autografts were used. If the graft diameter was less than 8 mm, one of the two grafts was tripled. After arthroscopic inspection of the patellofemoral joint and the cartilage and menisci of both compartments, the remaining ACL stumps were removed. First, the femoral socket was created through the anteromedial portal using a cannulated reamer 0.5 mm less than the diameter of the graft. Subsequently, the tibial tunnel was drilled using a cannulated reamer with the diameter of the graft. After introduction, the hamstring graft was fixated in the femoral socket with an endobutton (Endobutton CL Ultra; Smith & Nephew) and after 20 cycles the graft was fixated in the tibial tunnel with a plug and a peek interference screw (Biosure; Smith & Nephew) of 1 mm more than the diameter of the graft with the knee in 0–10 degrees of flexion.

### Study parameters

The primary outcome measures were the maximal ATTd during a single leg hop for distance (SLHD) landing determined using a passive motion capture system (VICON VERO; VICON Motion Systems Ltd., Oxford, UK) and its embedded methods and the MPTPA and LPTPA determined using MRI. The MRI’s were taken as part of the care as usual. In addition, primary study parameters during the jumping task were the knee flexion angle, and internal knee extension moment measured using a Vicon system and force platform (AMTI; Watertown, MA). During the single leg hop for distance surface electromyographic (sEMG) data was captured using Cometa electrodes (Cometa Wave Plus Wirless sEMG system, Cisliano Milano, Italy) of the medial hamstring (MH), lateral hamstring (LH), rectus femoris (RF), vastus medialis (VM), vastus lateralis (VL), gastrocnemius medialis (GM) and gastrocnemius lateralis (GL) [25]. A secondary study parameter was ATTp measured with the KT-1000 arthrometer (MEDmetric Corporation, San Diego, California, USA).

### Procedure

#### Dynamic ATT

**Fig. 1:**
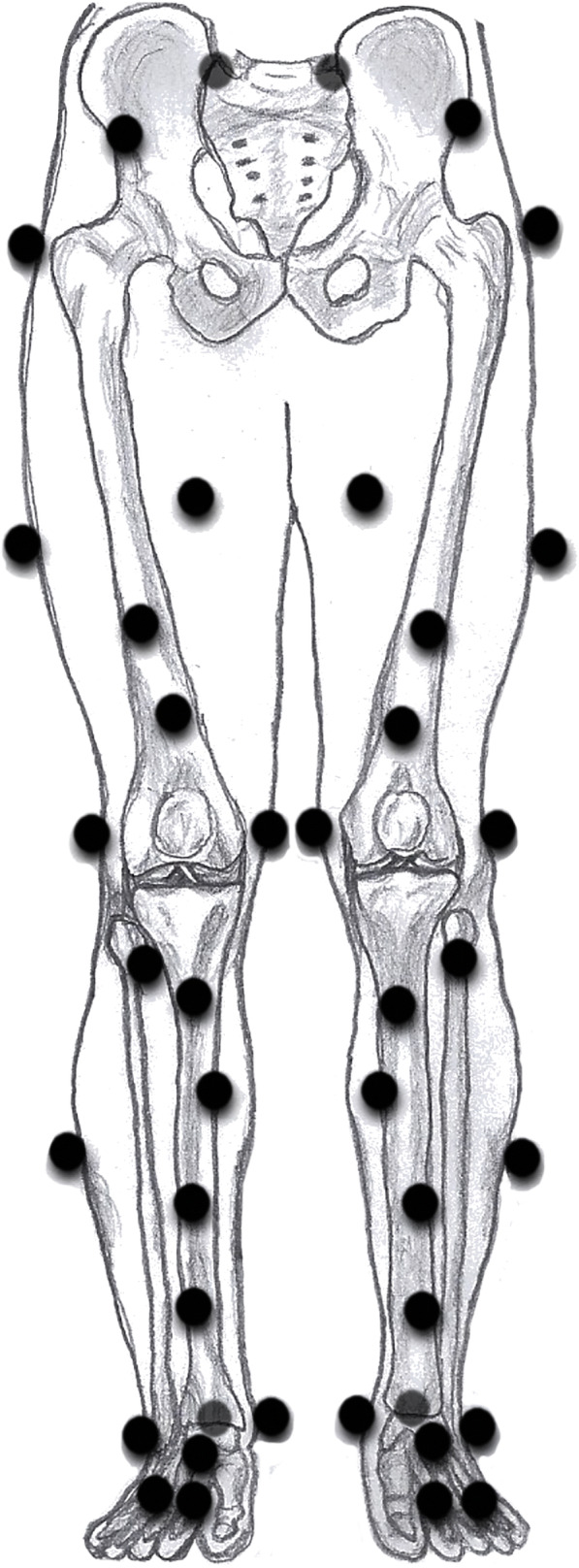
Marker placement. Markers were attached on the right and left anterior and posterior superior iliac spine, the right and left iliac crest, the greater trochanter, the medial and lateral epicondyles of the knee, the medial and lateral malleoli of the ankle, the heel, anterior of the talus bone and the first and fifth metatarsophalangeal joints. Besides, two additional markers were attached to the pelvis, two to the thigh, and six additional markers were attached to the shank (adapted from Boeth et al. [3])

First, 42 retroreflective markers and sEMG electrodes were attached to the participants. Markers were attached as shown in Fig. 1 (adapted from Boeth et al. [3]). sEMG electrodes were attached using SENIAM guidelines [25]. The same investigator (MNJK) performed all marker and electrode placements. Marker positions were measured using the 10-camera three-dimensional motion capture system Vicon at a frequency of 200 Hz. After attaching the markers, calibration frames of a flexion-extension movement and a star-arc movement, as prescribed by the manual of VICON, were performed to be able to identify the joint hip and knee centres and axes of rotation of the knees [8, 9]. Then, the SLHD were performed as described previously [35]. For another study the SLHD was performed for both legs, for this study only data of the operated leg was of interest. First, three practice jumps were performed with both legs. The participants hopped forwards as far as possible starting from standing still on their tested leg. They were instructed to stand still for at least 3 seconds after landing to assure a controlled landing. The median distance of the practice jumps was used as the starting distance from a 40 × 60 cm force platform (AMTI; Watertown, Massachusetts). Next, twenty successful jumps, 10 with each leg. Were performed where the participant landed on the force plate. The starting leg was randomised.

#### MPTPA and LPTPA using MRI

**Fig. 2:**
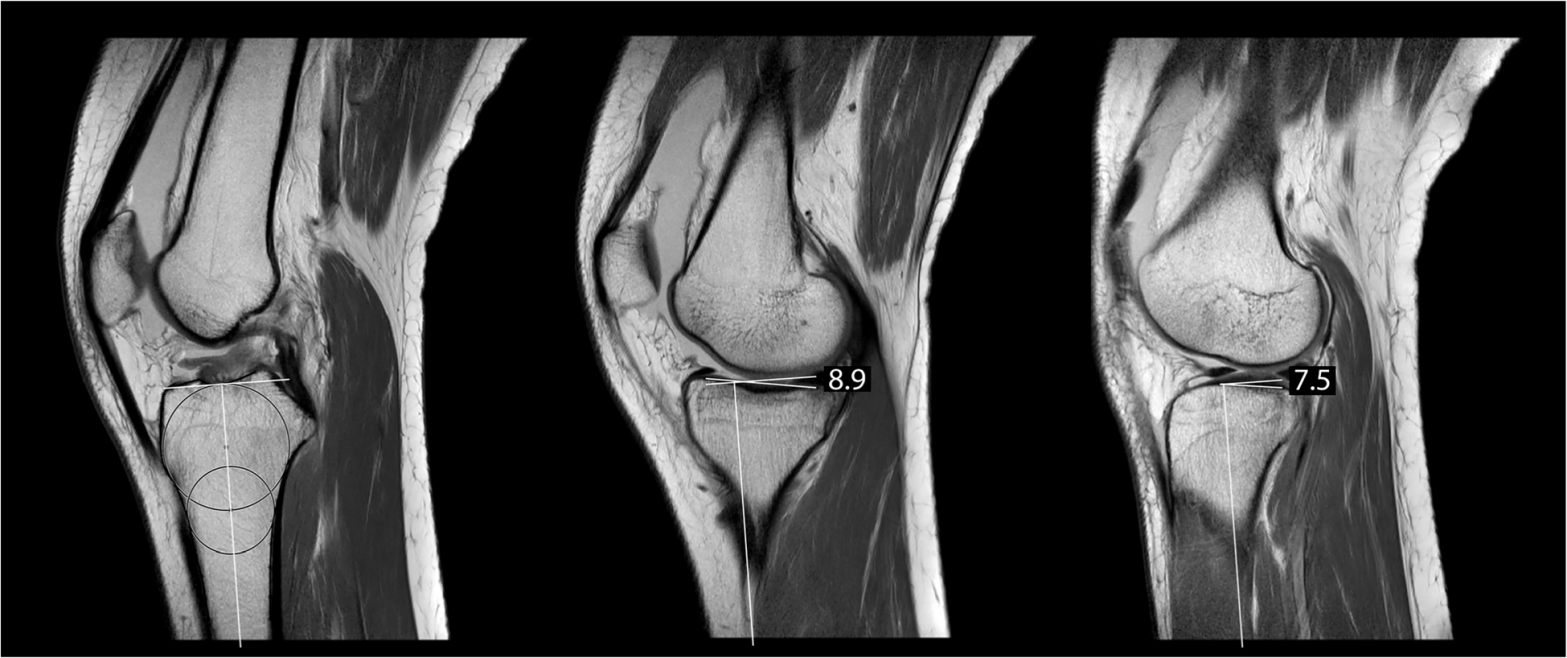
Determination of the medial and lateral PTPA using MRI and the circle method [12, 20]. First, the central sagittal MRI image was found (left image). This image was determined using the following criteria: the anterior and posterior proximal tibia cortices were visible in concave shape and the intercondylar eminence and the posterior cruciate ligament attachment were visible in the image. In this image, a circle was fitted to the proximal tibia, tangential to the cortices. A second circle was fitted distally in the tibia with its centre placed on the first circle. The longitudinal axis was determined by the line connecting the centres of the two circles. Then, the mid-sagittal images of the medial and lateral femoral condyles were selected (middle and right image). The angle between the line connecting the anterior and posterior articular surface of the posterior tibia plateau and the line at right angles to the longitudinal axis of the tibia on both medial and lateral images were the MPTPA and LPTPA respectively

The MPTPA and LPTPA were measured by means of MRI using the circle method [12, 20] using a customized MATLAB script. See Fig. 2 for a description of this procedure. This procedure was repeated three times for each slope and each participant by the same researcher. The mean of the three calculated angles was taken.

#### Quantifications of dynamic ATT and knee angles

For quantification of ATTd and knee angles see Keizer and Otten (2020). In brief, two coordinate systems were reconstructed in the tested knee using a customized MATLAB script based on the method of Boeth et al. [3]. One system was reconstructed in the femoral segment (parent system) and one in the tibia segment (child system). The motion of each coordinate system is consistent with the movement of the respective segment. After reconstruction of the two coordinate systems, the femoral coordinate system was translated and rotated towards the local tibia coordinate system. Finally, the anterior tibia translation was quantified in millimeters using the relative movement of the joint centre of rotation of the tibia coordinate system relative to the joint centre of rotation of the femoral coordinate system in the local tibial coordinate system. The coefficient of variation of this procedure across 16 healthy knees is 5.2% +/− 1.2% and excellent reproducibility was observed (ICC(3,1) = 0.92) [3]. Moreover, Keizer and Otten [17] showed that ATTd larger than 2.32 mm (mm) is reliable in terms of wobbling masses and the Vicon marker position error. The knee flexion angles, the rotations between both coordinate systems, tibia and femur, were calculated. The rotations are obtained using scalar products as in the equations explained in Robertson et al. [26].

### Data analysis

Data were processed and analysed using MATLAB version 9.4 (The MathWorks Inc., Natick, Massachusetts). The ATTd, sagittal knee angle and knee extension moment during each jump were determined from the moment of first ground contact until 0.5 s after the moment of first ground contact. The moment of first ground contact was determent as the moment where the vertical ground reaction force measured by the force plate was at least 5 % of the participants body weight expressed in N. Kinematic data were filtered using a convolution filter with low pass frequency of 10 Hz with zero lag. Using inverse dynamics, the ground reaction force vector and its lever arm to the centre of the knee of the stance leg where used to calculate the internal knee extension moment, which was normalized to body mass [38].

sEMG signals were recorded at 1000 Hz. The signals were rectify and a fourth-order low pass frequency Butterworth filter with a cut-off frequency of 6 Hz with zero lag was used to filter the muscle activity, taking into account an electromechanical delay of 50 milliseconds. The EMG signals were scaled to the mean muscle activity during 1 s before IC until 1.5 s after IC of the single leg hop for distance to minimize the effects of body fat and skin conductivity. The EMG was not scaled to the maximal muscle activity of an isometric contraction task as a large variation in peak activation of, especially the medial hamstrings muscle, during this task was observed.

### Statistical analysis

For all statistical analysis the Statistics Toolbox from MATLAB version 9.4 was used. To assess the intertrial repeatability of the approach to measure the MPTPA and LPTPA the intraclass correlation coefficients ICC(3,1) was determined [24].

A Pearson correlation was performed between ATTp and maximal ATTd. A multi-regression analysis with intercept was performed for both the MPTPA and LTPA correlating with the maximal ATTd, knee flexion angle and knee extension moment. A Pearson correlation was calculated between the maximal ATTd during jump landing and the MPTPA and between the maximal ATTd and the LPTPA. In addition, Pearson correlations were calculated between the MPTPA/LPTPA and the maximal knee moment, and between MPTPA/LPTPA and the maximal knee flexion angle. An alpha of ≤0.05 was considered to be significant. If a correlation was significant, a correlation coefficient of 0.2–0.49, 0.5–0.79 and 0.8–1 were considered to represent a weak, a moderate and a strong association, respectively [14].

A statistical parametric mapping (SPM) canonical correlation analysis (CCA) was performed to find the significance between muscle activity and the LPTPA or MPTPA over time. An open-source spm1d code (v.M.0.1, www.spm1d.org) in MATLAB version 9.7 was used to perform the SPM{X^2^}. For this analysis, data from initial contact until 0.5 s after initial contact was used. Correlations were calculated between LTPTA or the MPTPA and the activities of the muscles. The null hypothesis was rejected when the original SPM{X^2^} analysis exceeded the calculated critical X^2^-value (threshold) based on an alpha of ≤0.05, implying a correlation. When significant values were reached, a post-hoc regression analysis was performed over time for each muscle activity separately.

## Results

### MPTPA, LPTPA and ATT

The PTPA was measured three times for each participant by the same researcher. Excellent reproducibility was found between instances that the PTPA was calculated (MPTPA ICC(3,1) = 0.98, LPTPA ICC(3,1) = 0.98). For the mean and range of the MPTPA, LPTPA, ATTp and ATTd see Table 1. A non-significant correlation was found between ATTp and maximal ATTd (*p* = 0.20).

### Correlation between PTPA and kinematics

**Fig. 3:**
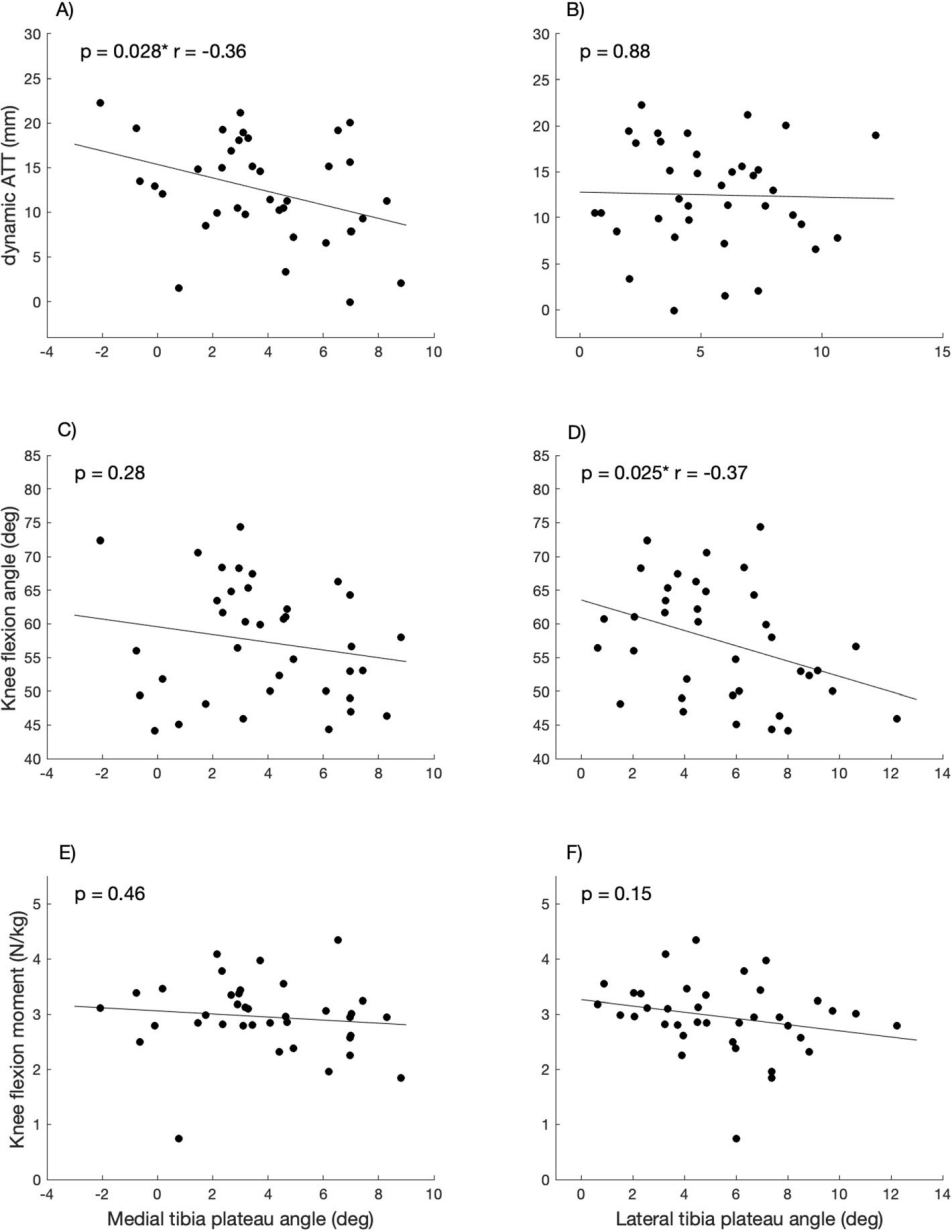
Correlations between MPTPA and the maximal dynamic ATT (**a**), knee flexion angle (**c**) and knee extension moment (**e**), and between LPTPA and the maximal dynamic ATT (**b**), knee flexion angle (**d**) and knee extension moment (**f**). *: significant

A multi-regression analysis with intercept revealed a non-significant correlation for the MPTPA (*p* = 0.18) and LPTPA (*p* = 0.09). See Table 2 and Fig. 3 for correlations between the tibia plateau angles, ATTd, maximal knee flexion angle and maximal knee extension moment.

**Table 2:** correlations between the tibia plateau angels and dynamic ATT, maximal knee flexion angles and maximal knee extension moments

		*p*-value	r-value
Dynamic ATT	MPTPA	0.028^a^	− 0.36
Dynamic ATT	LPTPA	0.88	N.S.
Maximal knee flexion angle	MPTPA	0.28	N.S.
Maximal knee flexion angle	LPTPA	0.025^a^	− 0.37
Maximal knee extension moment	MPTPA	0.46	N.S.
Maximal knee extension moment	LPTPA	0.15	N.S.
MPTPA	LPTPA	0.02^a^	0.38

^a^: significant

#### Muscle activation and PTPA

**Fig. 4:**
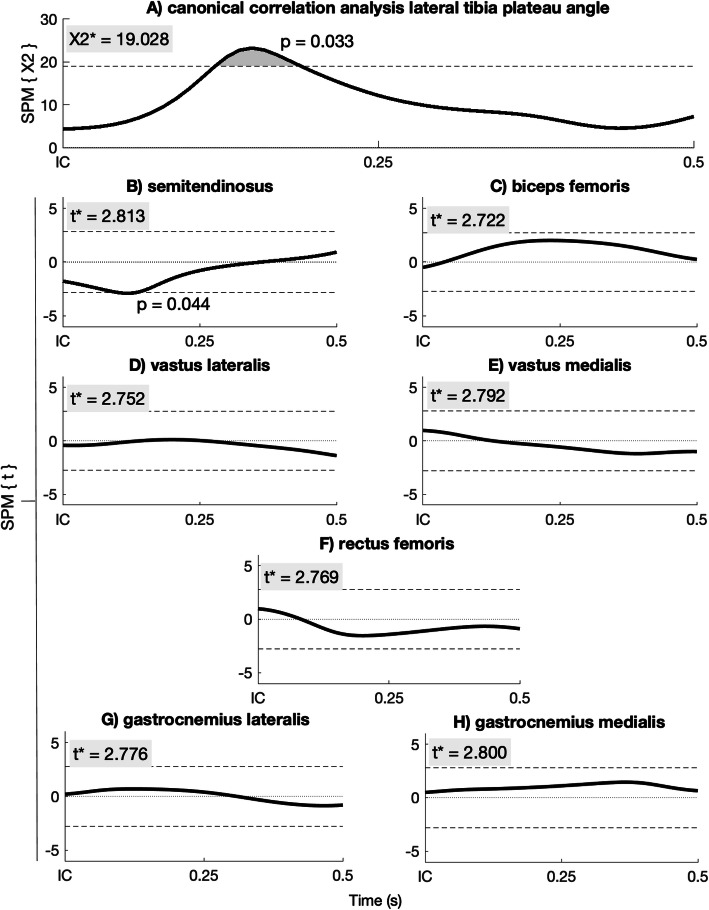
SPM{X^2^} canonical correlation analysis between muscle activity and the LPTPA (**a**). Pos-hoc SPM{t} regression analysis between LPTPA and the medial hamstrings (**b**), biceps femoris (**c**), vastus lateralis (**d**), vastus medialis (**e**), rectus femoris (**f**), gastrocnemius lateralis (**g**) and gastrocnemius medialis (**h**) activation. X2* and t* are the significant boundaries of the analysis

**Fig. 5:**
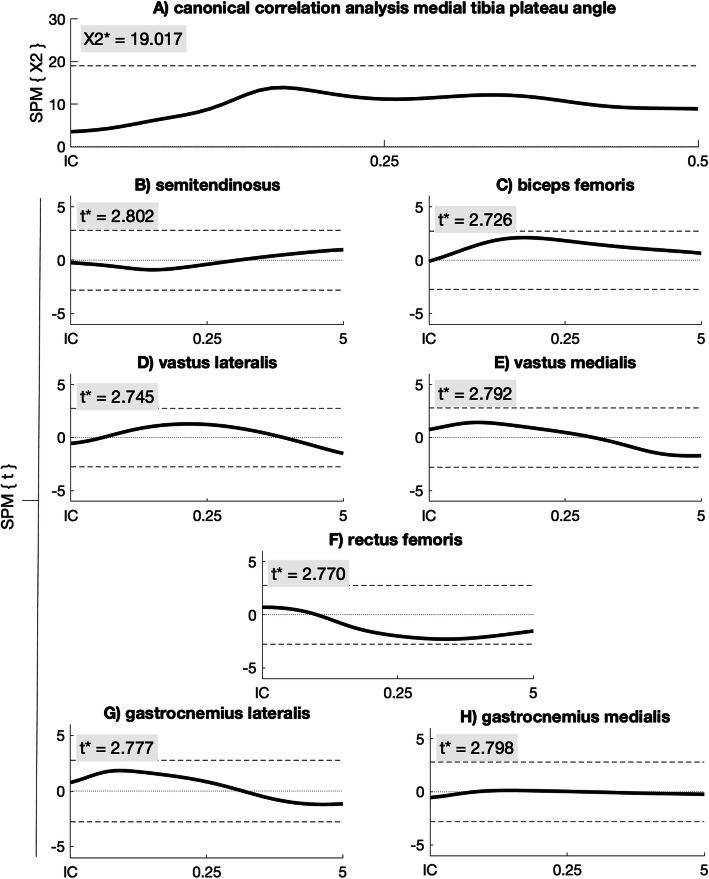
SPM{X^2^} canonical correlation analysis between muscle activity and the MPTPA (**a**). Pos-hoc SPM{t} regression analysis between MPTPA and the medial hamstrings (**b**), biceps femoris (**c**), vastus lateralis (**d**), vastus medialis (**e**), rectus femoris (**f**), gastrocnemius lateralis (**g**) and gastrocnemius medialis (**h**) activation. X2* and t* are the significant boundaries of the analysis

The SPM{X^2^} analysis of the lateral tibia plateau angle with the muscle activity showed a significant CCA between 6 and 10 ms after initial contact (*p* = 0.03; Fig. 4a). The post-hoc SPM{t} regression analysis showed a significant negative correlation of the medial hamstrings muscle between 4.5 and 7 ms after IC (Fig. 4b). All other muscles did not show a significant correlation with the lateral tibia plateau angle (Fig. 4c-h). The SPM{X^2^} analysis of the medial tibia plateau angle with the muscle activity showed a non-significant CCA (Fig. 5).

## Discussion

The most important findings of this study are that there is a weak significant negative correlation between ATTd and MPTPA and between the maximal knee flexion angle and LPTPA. Moreover, a significant correlation between muscle activation (especially a negative correlation of the medial hamstrings) and the LPTPA is found just after initial contact. This may imply that the slope of the PTPA is associated with the kinematics of ACL injured and reconstructed knees and that patients with a larger LPTPA automatically adapt their landing strategy (i.e. more knee flexion angle and medial hamstrings activity) to the anatomy of their knee.

Previous model studies showed that an increase in PTPA results in an increase in ATT (e.g. [30, 31]). Our study confirms that there is an association between ATTd during jump landing and the MPTPA. However, we found a negative correlation where the previous studies found a positive correlation: in our study, patients with larger MPTPA showed less maximal ATTd than patients with smaller MPTPA (r = − 0.36). This is not as we hypothesized. In a passive situation tibiofemoral contact force on the tibia plateau in people with a larger PTPA results in more ATTp than when the PTPA is smaller. One possible explanation for the contradiction may be that during dynamic situations compensational muscle activation patterns play a significant role in limiting the ATTd. A non-significant correlation between ATTp and ATTd also implies this. During our study, patients may have compensated for increased ATTd or PTPA by using suitable muscle activation patterns or kinematics. A significant CCA between muscle activation and the LPTPA just after initial contact is indeed found. This is in line with previous studies who found adaptative muscle activation patterns in patients after an ACL reconstruction during gait [27] and suggested that neuromuscular compensation strategies enable patients after an ACL reconstruction to return to high demanding sports activities [22]. A 3D computer model study fed with real in vivo data could investigate the effect of the PTPA on kinematics, kinetics and muscle activity in further detail.

It has been shown that patients who have had an ACL injury compared to a group that had no ACL injury have larger LPTPA [34]. In our study we found that patients after an ACLR with larger MPTPA showed less ATTd and did not find a significant correlation between the LPTPA and ATTd. The results of Sonnery-Cottet et al. [34] and our results seems to contradict each other. However, as described in the previous paragraph, patients after an ACLR may compensate for larger PTPA by using muscle activation patterns in a way to reduce ATTd. When there is a loss of feedforward muscular activation control, for instance at foot positions that occur in a very short time span, an ACL (re)injury may occur in patients with a large PTPA because forces are not carried by the muscular components. This is in line with previous studies that showed that the PTPA correlates with an increased ATTp in ACL injured, ACL reconstructed and cadaveric knees [5, 13, 28]. We did find a significant CCA correlation between the LPTPA and muscle activation, which suggests that patients indeed amend their muscle activation to the anatomy of their knee, especially by reducing their medial hamstring activity when the LPTPA is larger. This result is in line with our finding that patients with larger LPTPA showed smaller maximal knee flexion angle during jump landing than patients with smaller LPTPA. Patients with a larger LPTPA may automatically adapt their landing strategy (i.e. maximal knee flexion angle and hamstring activation) to their anatomy of the knee. Less knee flexion angle reduces ATTd. Further research could investigate if patients with larger PTPA use different muscle activation patterns than patients with smaller PTPA to reduce the ATTd.

The MPTPA and LPTPA have a low correlation: some patients with large MPTPA have small LPTPA (the difference between MPTPA and LPTPA is − 9.1 to 3.7 (mean: − 1.82) degrees in our study). Previous studies found asymmetry (a nonsignificant or weak correlation) between the LPTPA and MPTPA [11, 23, 37]. A large LPTPA does not necessarily imply that these patients also have a large MPTPA. Our results showed, in most cases, a larger LPTPA than the MPTPA, which is also found in the study of Hashemi et al. [11]. Hashemi et al. [11] showed a range of MPTPA of − 3 to 10 degrees (our study − 2.1 to 8.8) and of LPTPA of 0 to 14 degrees (our study 0.6 to 12.2). We suggest that a larger LPTPA than the MPTPA is beneficial in terms of biomechanics because this combination provides a larger internal tibia rotation moment during walking. A larger internal tibia rotation moment (counteracted by a moment from the floor) helps to rotate the trunk in swinging the other leg forwards during walking. Also, our results showed a correlation between ATTd and MPTPA, however, not between ATTd and LPTPA.

Some orthopaedic surgeons even consider and recommend a combined ACLR and anterior closing wedge tibial osteotomy in patients after an ACL injury in order to reduce the PTPA [6, 7, 13]. It is shown that with an absence of applied internal moment, an anterior closing wedge tibial osteotomy in cadaveric knees alters knee kinematics, reduced the ATTp, which results in a reduction of ACL load [13, 39]. However, neuromuscular control, kinematics and kinetics were not taken into account. Future studies should investigate whether the ATTd is reduced, in situations with loss of muscular activation control, after an anterior closing wedge tibial osteotomy to confirm this suggestion.

### Limitations

A limitation of this study that needs to be addressed is the potential influence of wobbling masses on the measured ATTd. However, Keizer and Otten [17] have identified the sensitivity of the method to determine ATTd on the marker placement (wobbling masses) and Vicon’s position error. The error found in this sensitivity study was less than 2.32 mm [17]. The results of the current study were interpret using this error. A lack of golden standard makes it impossible to verify the outcomes of the methods used. When compared to previous studies the range of ATTd is comparable. In previous studies the ATTd range was 11.5 mm (− 4.7 to 6.8 mm) [16] and 12 mm (− 2 to 10 mm) [17] both using the same methods, 10 mm (8 to 18 mm) using bi-planar fluoroscopy model-based data during running [1]; all in healthy subjects. In our study the mean range of ATTd was 13.42 (− 2.96 to 10.46). A second limitation may be the method of normalization of muscle activity. Muscle activation was normalized to the percentage of the mean muscle activity during the single leg hop for distance. This normalized muscle activation may be more comparable between patients than the absolute maximal electrical muscle activation. This was done because we observed large differences in the maximal muscle activation between legs during this task, especially in the medial hamstrings muscle. Since we are averaging over subjects in a group, this provides numerical more stable results. A third limitation is the absence of information about the tibia plateau slope of the contralateral knee. It would be very interesting for future study to investigate the contralateral tibia plateau slope in correlation with muscle activation and the difference between the contralateral and injured knee.

## Conclusion

The ATTd during a single leg hop for distance is negatively correlated with the MPTPA, however not with the LPTPA. Moreover, patients with a smaller LPTPA show larger maximal knee flexion angle during landing than the patients with larger LPTPA. Moreover, a correlation between the LPTPA and semitendinosus activity was found.

## Data Availability

The datasets used and/or analysed during the current study are available from the corresponding author on reasonable request.

## Abbreviations

ATTd: dynamic anterior tibia translation
ATTp: passive anterior tibia translation
ACL: anterior cruciate ligament
MPTPA: medial posterior tibia plateau angle
LPTPA: lateral posterior tibia plateau angle
SLHD: single leg hop for distance
ICC: intraclass correlation coefficient

## References

[1] Anderst W, Zauel R, Bishop J, Demps E, Tashman S (2009) Validation of three-dimensional model-based Tibio-femoral tracking during running. Med Eng Phys 31(1):10-6 10.1016/j.medengphy.2008.03.003 PMC2668117 18434230

[2] Bates NA, Nesbitt RJ, Shearn JT, Myer GD, Hewett TE, Hewett TE (2016). Posterior Tibial slope angle correlates with peak sagittal and frontal plane knee joint loading during robotic simulations of athletic tasks. Am J Sports Med.

[3] Boeth H, Duda GN, Heller MO, Ehrig RM, Doyscher R, Jung T, Moewis P, Scheffler S, Taylor WR (2013). Anterior cruciate ligament-deficient patients with passive knee joint laxity have a decreased range of anterior-posterior motion during active movements. Am J Sports Med.

[4] Butler DL, Noyes FR, Grood ES (1980). Ligamentous restraints to anterior-posterior drawer in the human knee. A biomechanical study. J Bone Jt Surg - Ser A.

[5] Dejour D, Pungitore M, Valluy J, Nover L, Saffarini M, Demey G (2019) Tibial slope and medial meniscectomy significantly influence short-term knee laxity following ACL reconstruction. Knee Surg Sports Traumatol Arthrosc. 27(11):3481-3489 10.1007/s00167-019-05435-0 30809722

[6] Dejour D, Saffarini M, Demey G, Baverel L (2015). Tibial slope correction combined with second revision ACL produces good knee stability and prevents graft rupture. Knee Surgery, Sport Traumatol Arthrosc.

[7] DePhillipo NN, Kennedy MI, Dekker TJ, Aman ZS, Grantham WJ, LaPrade RF (2019). Anterior closing wedge proximal Tibial osteotomy for slope correction in failed ACL reconstructions. Arthrosc Tech.

[8] Ehrig RM, Taylor WR, Duda GN, Heller MO (2006). A survey of formal methods for determining the Centre of rotation of ball joints. J Biomech.

[9] Ehrig RM, Taylor WR, Duda GN, Heller MO (2007). A survey of formal methods for determining functional joint axes. J Biomech.

[10] Grassi A, Signorelli C, Urrizola F, Macchiarola L, Raggi F, Mosca M, Samuelsson K, Zaffagnini S (2019) Patients with failed anterior cruciate ligament reconstruction have an increased posterior lateral Tibial plateau slope: a case-controlled study. Arthroscopy 35(4):1172-1182 10.1016/j.arthro.2018.11.049 30878331

[11] Hashemi J, Chandrashekar N, Gill B, Beynnon BD, Slauterbeck JR, Schutt RC Mansouri H. Dabezies E The Geometry of the Tibial Plateau and Its Influence on the Biomechanics of the Tibiofemoral Joint. J Bone Joint Surg Am 90(12):2724-34 10.2106/JBJS.G.01358 PMC2663332 19047719

[12] Hudek R, Schmutz S, Regenfelder F, Fuchs B, Koch PP (2009). Novel measurement technique of the tibial slope on conventional MRI. Clin Orthop Relat Res.

[13] Imhoff FB, Mehl J, Comer BJ, Obopilwe E, Cote MP, Feucht MJ, Wylie JD, Imhoff AB, Arciero RA, Beitzel K (2019) Slope-reducing tibial osteotomy decreases ACL-graft forces and anterior tibial translation under axial load.Knee Surg Sports Traumatol Arthrosc 27(10):3381-3389 10.1007/s00167-019-05360-2 30687890

[14] Jacob C (1988). Statistical power analysis for the behavioral sciences.

[15] Jaecker V, Drouven S, Naendrup JH, Kanakamedala AC, Pfeiffer T, Shafizadeh S (2018). Increased medial and lateral tibial posterior slopes are independent risk factors for graft failure following ACL reconstruction. Arch Orthop Trauma Surg.

[16] Keizer MNJ, Hijmans JM, Gokeler A, Benjaminse A, Otten E (2020) Healthy subjects with lax knees use less knee flexion rather than muscle control tolimit anterior tibia translation during landing. J Exp Orthop 15;7(1):32 10.1186/s40634-020-00246-6 PMC7229106 32415565

[17] Keizer MNJ, Otten E (2020) Technical note: sensitivity analysis of the SCoRE and SARA methods for determining rotational axes during tibiofemoral movements using optical motion capture. J Exp Orthop 7(1):6 10.1186/s40634-020-0219-z PMC7010897 32040787

[18] Kızılgöz V, Sivrioğlu AK, Ulusoy GR, Yıldız K, Aydın H, Çetin T (2019). Posterior tibial slope measurement on lateral knee radiographs as a risk factor of anterior cruciate ligament injury: a cross-sectional study. Radiography.

[19] Li Y, Hong L, Feng H, Wang Q, Zhang J, Song G, Chen X, Zhuo H (2014). Posterior tibial slope influences static anterior tibial translation in anterior cruciate ligament reconstruction: a minimum 2-year follow-up study. Am J Sports Med.

[20] Lipps DB, Wilson AM, Ashton-Miller JA, Wojtys EM (2012). Evaluation of different methods for measuring lateral Tibial slope using magnetic resonance imaging. Am J Sports Med.

[21] Navacchia A, Bates NA, Schilaty ND, Krych AJ, Hewett TE (2019). Knee abduction and internal rotation moments increase ACL force during landing through the posterior slope of the tibia. J Orthop Res.

[22] Nyland J, Mauser N, Caborn DNM (2013). Sports involvement following ACL reconstruction is related to lower extremity neuromuscular adaptations, subjective knee function and health locus of control. Knee Surgery, Sport Traumatol Arthrosc.

[23] Pei Yuik Ho J, Merican AM, Sufian Hashim M, Abbas AA, Ken Chan C, Mohamad JA (2017) Three-Dimensional Computed Tomography Analysis of the Posterior Tibial Slope in 100 Knees. J Arthroplasty 32(10):3176-3183 10.1016/j.arth.2017.04.060 28579444

[24] Portney LG, Watkins MP (2009). Foundations of clinical research: applications to practice.

[25] Project TS (2016). Surface ElectroMyoGraphy for the non-invasive assessment of muscles.

[26] Robertson G, Galdwell G, Hamill J, Kamen G, Whittlesey S (2013). Research methods in biomechanics, 2E.

[27] Roper JA, Terza MJ, Tillman MD, Hass CJ Adaptation Strategies of Individuals With Anterior Cruciate Ligament Reconstruction. Orthop J Sports Med 8;4(2):2325967115627611 10.1177/2325967115627611 PMC4748157 26894200

[28] Sauer S, Clatworthy M (2018). The effect of medial Tibial slope on anterior Tibial translation and short-term ACL reconstruction outcome. Surg J.

[29] Sayit E, Sayit AT, Terzi Y (2017). Evaluation of the posterior Tibial slope in noncontact ACL injuries using magnetic resonance imaging. Acta Orthop Belg.

[30] Shao Q, MacLeod TD, Manal K, Buchanan TS (2011). Estimation of ligament loading and anterior tibial translation in healthy and ACL-deficient knees during gait and the influence of increasing tibial slope using EMG-driven approach. Ann Biomed Eng.

[31] Shelburne KB, Kim HJ, Sterett WI, Pandy MG (2011). Effect of posterior tibial slope on knee biomechanics during functional activity. J Orthop Res.

[32] Shelburne KB, Pandy MG (2010) A dynamic model of the knee and lower limb for simulating rising movements. Comput Methods Biomech Biomed Engin 5(2):149-59 10.1080/10255840290010265 12186724

[33] Shen X, Xiao J, Yang Y, Liu T, Chen S, Gao Z, Zuo J (2019) Multivariable analysis of anatomic risk factors for anterior cruciate ligament injury in active individuals. Arch Orthop Trauma Surg 139(9):1277-1285 10.1007/s00402-019-03210-x 31190114

[34] Sonnery-Cottet B, Archbold P, Cucurulo T, Fayard J-M, Bortolletto J, Thaunat M, Prost T, Chambat P, Archbold P, Surgeon O, Bortolletto J, Cucurulo T (2011). The influence of the tibial slope and the size of the intercondylar notch on rupture of the anterior cruciate ligament. J Bone Jt Surg Br.

[35] VDHarst JJ, Gokeler A, Hof AL (2007) Leg kinematics and kinetics in landing from a single-leg hop for distance. A comparison between dominant and non-dominant leg. Clin Biomech 22:674–680 10.1016/j.clinbiomech.2007.02.007 17418922

[36] Webb JM, Salmon LJ, Leclerc E, Pinczewski LA, Roe JP (2013). Posterior tibial slope and further anterior cruciate ligament injuries in the anterior cruciate ligament-reconstructed patient. Am J Sports Med.

[37] Differences in Medial and Lateral Posterior Tibial Slope An Osteological Review of 1090 Tibiae Comparing Age, Sex, and Race. Am J Sports Med 45(1):106-113 10.1177/0363546516662449 27587744

[38] Winter DA. (2009) Biomechanics and Motor Control of Human Movement. 4th Edition, Wiley, Hoboken.

[39] Yamaguchi KT, Cheung E, Mathew J, Boguszewski DV, Markolf K Mcallister DR. Petrigliano FA ACL Force and Knee Kinematics After Posterior Tibial Slope-Reducing Osteotomy. Am J Sports Med 46(2):370-377 10.1177/0363546517736767 29100001

